# Black Chokeberry Extracts (*Aronia melanocarpa*) as an Ingredient of Functional Food—Potential, Challenges and Directions of Development

**DOI:** 10.3390/molecules30214237

**Published:** 2025-10-30

**Authors:** Dawid Wieloch, Dorota Konopacka

**Affiliations:** HortiFood Processing Centre, Fruit and Vegetable Storage and Processing Department, The National Institute of Horticultural Research, Rybickiego 15/17, 96-100 Skierniewice, Poland; dorota.konopacka@inhort.pl

**Keywords:** green extracts, chokeberry, functional food, polyphenols, anthocyanins

## Abstract

Functional food is gaining global importance as consumer demand for products delivering health benefits beyond basic nutrition increases. Black chokeberry (*Aronia melanocarpa*) is a promising candidate in this field, due to its exceptionally high content of bioactive compounds, particularly polyphenols with well-documented health-promoting properties. This article reviews the current state of knowledge about the functional food definition and the health benefits of chokeberries, with special emphasis given to their extracts as promising ingredients for novel product development. Efficient recovery methods for bioactive compounds from fruits, pomace, and leaves were discussed, including advances in green extraction technologies such as ultrasound- and microwave-assisted extraction, supercritical fluid extraction and enzyme-assisted extraction. Stabilization approaches, including microencapsulation and freeze-drying, which enhance the stability and bioavailability of phenolics, were also highlighted. The impact of aronia extracts on technological and sensory parameters of food was investigated. Applications in beverages, baked goods, dairy, and meat products demonstrate improved antioxidant capacity and storability. However, astringency remains a major sensory challenge. Future perspectives include optimization of processing strategies and developing synergistic formulations to maximize health benefits while ensuring consumer acceptance.

## 1. Introduction

From the consumer’s perspective, functional food products in addition to their basic nutritional value can provide an additional, beneficial effect on the human body—supporting its functioning or reducing the risk of developing noncommunicable diseases. Growing consumer nutritional awareness, as well as technological advances in the food industry, are contributing to the dynamic development of this product segment. One way to obtain products with exceptional health-promoting properties is to enrich traditional raw materials with bioactive phytochemical compounds, e.g., by adding plant extracts [1,2,3,4]. In this context, black chokeberry (*Aronia melanocarpa*), also recognized as aronia, deserves special attention, as its exceptionally high content of polyphenols, including anthocyanins, flavonoids and phenolic acids, which give strong antioxidant and anti-inflammatory properties. Due to the high content of bioactives, a beneficial effect on the health of the cardiovascular system was detected [5,6,7]. Aronia extracts are available in the form of juice concentrates, powders, water-alcoholic extracts, pomace and others, constituting a valuable functional ingredient that can be used in a wide range of food products. However, introducing products significantly enriched with aronia extracts into to the consumer market is associated with technological, sensory and regulatory challenges that must be considered in the process of designing functional food [8,9,10,11].

The aim of this article was to discuss the potential of black chokeberry extracts as a component of functional food, to point out key barriers to their implementation, as well as to identify possible directions for further development in this area.

## 2. Functional Food—Definitions and Market Significance

The first definition of functional food comes from Japan. Research on this group of products began in 1984. In 1991, the Minister of Health of Japan approved a new food category called FOSHU (Food for Specified Health Use). As a result of the Japanese government designating this type of product as a separate assortment, legislative work on the legal definition of functional food has begun both in Europe and in the USA. Despite more than three decades of discussion, no agreement has been reached on how to define and regulate the term. Different scientific and governmental institutions use different definitions of functional food, which differ from each other in terms of the adopted criteria and are not mutually identical. The most frequently quoted definition of functional food in Europe is the one developed under the Functional Food Science in Europe (FUFOSE) programme, coordinated by the International Life Sciences Institute (ILSI): “A product can be considered functional only if, at the same time as the basic nutritional value, it exerts an additional effect on one or more functions of the human body, both by improving general and physical conditions and/or reducing the risk of disease development. The amount of intake and form of functional food should be what is normally expected for nutritional purposes. Therefore, it cannot be in the form of pills or capsules, but in the form of normal food” [12,13,14,15]. In the United States, a new concept of defining the concept of “functional food” emerged in 1999. Researchers from the Functional Food Center and representatives of the Food and Drug Administration (FDA) and the Academic Society of Functional Foods and Bioactive Compounds (ASFFBC), in cooperation with the United States government, defined “functional food” as: “natural or processed foods that contain biologically active compounds that, in specific, effective, non-toxic amounts, provide clinically proven and documented health benefits using specific biomarkers, to promote optimal health and reduce the risk of chronic/viral diseases and manage their symptoms’’ [16,17].

According to the latest Global Market Insights report [18], the functional food segment will experience exceptionally dynamic growth. The compound annual growth rate (CAGR) for 2025–2034 is projected at 8–12%. This trend is driven by the growing interest in healthy lifestyles by consumers, who are increasingly making food and purchasing choices based on information about product composition and their impact on health [19,20,21,22]. The fact that the population is ageing and the related problem of escalating chronic diseases, which generate huge costs of medical care, are also important. This prompts both governments and international organizations to support all activities and regulations that may have a real impact on extending healthy life expectancy [23]. This creates great development opportunities for the food sector, and in particular for the functional food market.

Within this broader context, the development and commercialization of black chokeberry (*Aronia melanocarpa*) products could strongly benefit from current market trends. Increasing consumer awareness of the health benefits associated with natural antioxidants has already boosted the popularity of chokeberry-based foods and beverages [24]. The fruit’s exceptionally high content of polyphenols, anthocyanins, and other bioactive compounds aligns well with the growing consumer preference for plant-derived ingredients that provide measurable health-promoting effects. Consequently, industrial interest has shifted towards the extraction and concentration of bioactive compounds from black chokeberry, aimed at producing high-potency extracts for use as functional ingredients in various food matrices or as the basis for formulations exhibiting exceptionally strong antioxidant activity.

## 3. Black Chokeberry—Health-Promoting Properties and Bioactive Composition

In the process of designing new food products from the functional food category, it is important to select raw materials with documented health-promoting properties, going beyond the standard nutritional value typical for raw materials of a given category. An example of such a fruit is the black chokeberry (*Aronia melanocarpa*), belonging to the *Rosaceae* family. Aronia is a fruit with a high content of bioactive compounds, primarily polyphenols, anthocyanins, flavonoids and phenolic acids [25,26,27], which are responsible for its broad spectrum of health-promoting properties. With regard to other pro-health properties *Aronia* also contains significant amounts of dietary fibre, sorbitol and some ascorbic acid. The scientific literature in this field is very rich [28,29,30,31]. Table 1 presents the major bioactive compounds of black chokeberry (*Aronia melanocarpa* L.) and their associated biological effects.

Systematic reviews of the literature indicate the beneficial effect of aronia supplementation on the reduction in inflammation and oxidative stress in humans and animals. In clinical trials, a decrease in the levels of pro-inflammatory cytokines such as IL-6, TNF-α and CRP was observed, as well as an increase in the anti-inflammatory interleukin IL-10. In addition, aronia supplementation improved the activity of antioxidant enzymes, among other superoxide dismutase (SOD), catalase (CAT) and glutathione peroxidase (GSH-Px), which confirms its role in the modulation of the immune system and antioxidant protection of the body [39,40,41,42].

In studies on animal models, aronia extracts have shown encouraging results in lowering uric acid levels, inhibiting xanthine oxidase activity, and reducing oxidative stress markers such as malondialdehyde, while increasing glutathione levels—indicating strong antioxidant activity and kidney protection in mice with hyperuricemia. These effects were comparable to those of the drug allopurinol, highlighting the potential of aronia as a natural functional agent in the treatment of hyperuricemia and related metabolic disorders [43,44,45,46].

Research also indicates the neuroprotective properties of aronia. Aronia extracts have demonstrated an ability to inhibit inflammatory processes in microglial cells and protect neurons against amyloid beta-induced apoptosis, which suggests potential in the prevention and treatment of neurodegenerative diseases such as Alzheimer’s disease [47]. These mechanisms include modulation of the expression of genes associated with apoptosis, reduction in oxidative stress, and improvement of mitochondrial function [48,49,50,51].

With a high content of anthocyanins and polyphenols, the cardioprotective effects of aronia were documented [52,53,54]. Surprisingly, the results of clinical trials in this regard are ambiguous. Meta-analysis of randomized controlled trials showed that aronia supplementation did not provide significant benefits in terms of cardiometabolic parameters in the general population. However, in selected subgroups, such as those with lower total cholesterol levels or at doses of anthocyanins above 50 mg/day, a beneficial effect was found to lower LDL cholesterol and systolic blood pressure [55]. Therefore, further well-designed studies are needed to confirm these observations [56].

As the latest clinical research results cited above indicate that the black chokeberry is a promising ingredient for creating the functional food. Nevertheless, a product formulated with aronia or aronia extracts demonstrate health-promoting effects, the processing methods used, require extensive optimization, facilitated by technological advancements.

## 4. Modern Techniques for Obtaining Plant Extracts

Recent years have brought rapid advances in extraction technologies aimed at improving the recovery, purity, and stability of plant bioactive compounds. In addition to enhancing process efficiency and preserving the chemical integrity of the extracted compounds, modern techniques also focus on reducing energy consumption, minimizing the use of synthetic solvents, and ensuring overall process sustainability.

This section discusses the most important innovative methods for obtaining plant extracts relevant to berry fruits, with particular emphasis on their potential applications in the design of functional foods.

### 4.1. Ultrasound-Assisted Extraction

Ultrasound-Assisted Extraction (UAE) is a technique that uses ultrasonic waves at 20–100 kHz to induce the phenomenon of cavitation in an extraction liquid. Cavitation leads to the formation of gas bubbles, which, when imploding in the vicinity of plant particles, causes mechanical damage to cell walls and thus facilitates the diffusion of bioactive compounds into the solvent [57]. This technology is characterized by high efficiency, low solvent consumption and short process time. UAE is particularly useful for the extraction of phenolic compounds, flavonoids and anthocyanins from fragile raw materials such as fruits, leaves or flowers [58]. The reduced extraction time and moderate temperatures are crucial for preserving thermolabile phytochemicals, making this especially advantageous for heat-sensitive bioactive compounds. For the extraction of polyphenolic compounds from most plant matrices, a 30–40 min treatment at 40 kHz and 40–60 °C has been reported to provide satisfactory recovery yields [59].

### 4.2. Microwave-Assisted Extraction

Microwave-Assisted Extraction (MAE) involves dielectric heating of the plant raw material using microwave radiation with a frequency from 0.3 to 300 GHz, mainly at 2.45 GHz. Microwave energy induces a rapid rise in temperature and pressure within plant cells, provided that the selected solvent has a high dielectric constant and efficiently absorbs microwave radiation. Solvents such as ethanol, methanol and water are sufficiently polar to be heated by microwave energy, thus making the technique suitable for extraction of bioactive such as wide range of polyphenols. MAE offers a rapid delivery of energy to a total volume of solvent and solid plant matrix with subsequent heating of the solvent and solid matrix, efficiently and homogeneously. Internal superheating leads to cell wall disruption and release of active ingredients into the liquid phase. Although in the case of the extraction of thermolabile compounds, high temperatures may cause the degradation of extracts. As the process is really rapid, the final losses are relatively low. For example the extraction of tea polyphenols and caffeine from green tea leaves, in 4 min MAE achieved a higher extraction yield than an extraction at room temperature for 20 h or ultrasonic extraction for 90 min [58]. In summary, compared to traditional solvent extraction techniques, MAE is rapid, uses less solvent, is economical, and enables higher extraction yield [59].

### 4.3. Supercritical Fluid Extraction

Supercritical Fluid Extraction (SFE) is based on the use of supercritical carbon dioxide (CO_2_) (above 31 °C and 73 atm.) as a solvent. In this state, CO_2_ exhibits both liquid and gas properties, which enables the effective dissolution of non-polar components. Due to the low process temperature and the absence of residual solvents, this method is best-suited for the production of high-quality food extracts, including extraction of thermally labile compounds and is widely used in both laboratory and industrial applications [60].

### 4.4. Accelerated Solvent Extraction

Pressurized Liquid Extraction (or Accelerated Solvent Extraction) (PLE/ASE) is a technique that involves the use of an extraction liquid at elevated temperatures and pressures. Such conditions improve the solubility and diffusion rate of bioactive compounds. The closed system in which this technique is performed results in both the time saving and the efficient extraction of polar compounds, such as phenols or glycosides, from a wide range of plant based raw materials. The advantage of this method is the possibility of using friendly solvents (e.g., ethanol, water) and the reduction in process time compared to conventional techniques [61].

### 4.5. Extraction with Natural Eutectic Liquids

Natural Deep Eutectic Solvents (NADES) are biodegradable mixtures of natural organic compounds, such as amino acids, sugars or organic acids, which in the appropriate proportions can form liquid systems with enhanced solvent properties. NADES are non-toxic and therefore allow to classify the extract as “green-labelled” functional food. The latest research indicated the ability of NADESs towards selective extraction of phenolics: anthocyanins, flavonoids and phenolic acids from berry fruit, including aronia. The technique is considered a highly promising approach for the sustainable production of dietary supplements and functional food [60].

### 4.6. Enzyme-Assisted Extraction

Enzyme-Assisted Extraction (EAE) uses hydrolytic enzymes such as pectinase, cellulase and hemicellulase to degrade the cellular structure of plant material. These enzymes facilitate the release of active ingredients, especially from fibre-rich tissues or cell walls that are difficult to break mechanically, such as leaves or seed husks. EAE is a benign technique, carried out at low temperatures, which reduces the degradation of thermolabile compounds. The enzyme concentration and pH vary depending on the enzymes’ nature and action. The EAE is usually utilized in conjunction with other extraction techniques as the enzymes make non-extractable phytochemicals accessible to the solvent and hence vulnerable for extraction [59,62].

### 4.7. Pulsed Electric Field

Pulsed Electric Field (PEF) is a non-thermal technique in which short-term high-voltage pulses (microseconds to milliseconds intervals) are applied in order to induce electroporation of cell membranes. Electric field intensity above critical limits (0.8–1 V) leads to changes in the cellular structure, creating pores which facilitate diffusion of intracellular material. Thus, PEF allows cell permeability to be increased and thus facilitates the release of secondary metabolites into the solvent. This technique is increasingly used as a pre-step before actual extraction (e.g., UAE, IAE, SFE) [63].

### 4.8. Hybrid Extractions and an Integrated Approach

Contemporary research on the extraction of bioactive compounds from plant materials is increasingly shifting towards combined (“hybrid”) approaches that merge the advantages of different techniques to maximize yield, enhance extract quality, and ensure more efficient utilization of raw materials, including post-production residues. Examples of such hybrid strategies include coupling ultrasound-assisted extraction (UAE) with natural deep eutectic solvents (NADES), microwave-assisted extraction (MAE) with enzymatic cell wall degradation, and pulsed electric field (PEF) treatment combined with supercritical fluid extraction (SFE). These integrated methods not only shorten extraction time and improve recovery efficiency but also align with the principles of the circular economy by enabling the valorization of secondary materials (e.g., chokeberry pomace) and reducing both energy and solvent consumption.

In the case of berry fruits, where bioactive compounds are often tightly bound to the tissue matrix and enclosed within the intact skin, hybrid extraction approaches appear particularly advantageous. The combined application of techniques can offer additive or even synergistic benefits—for example, by enhancing the disruption of fruit tissue structure, improving solvent penetration, lowering the required process temperature, and thus reducing the degradation of thermolabile bioactive compounds [64,65].

Nevertheless, a review of the literature indicates that while numerous studies have focused on individual extraction techniques for chokeberry (e.g., ultrasound-assisted extraction, microwave-assisted extraction, enzymatic treatments), research exploring the integration of multiple methods remains scarce. One of the few notable exceptions is the “extraction–adsorption” process proposed by Galván D’Alessandro et al., in which chokeberry extraction was coupled with the use of an adsorption resin (XAD-7HP) in a single step. This integrated approach achieved up to 82% recovery of total polyphenols and up to 92% of anthocyanins—a remarkable improvement compared with non-hybrid extraction methods [66].

## 5. Stabilization of Extracts Using Microencapsulation

Plant extracts obtained using the advanced techniques described above, particularly those rich in anthocyanins and phenolic acids, are highly sensitive to oxygen, light, elevated temperature, or extreme pH changes. To preserve the benefits achieved through high-yield and selective extraction methods of bioactive compounds, it is essential to apply stabilization techniques. Among them, microencapsulation has become one of the most widely used approaches, as it allows bioactive constituents to be embedded within a protective matrix that shields them from degradation and loss of functionality.

In practice, encapsulation serves as the final stage of the technological process: it increases the concentration of active compounds, enables the conversion of liquid extracts into powder form, reduces pigment and antioxidant degradation. The protective matrix prevents losses during processing and storage, resulting in a higher content of the active ingredient in the finished product. In addition, microencapsulation may improve the dispersion and solubility of poorly soluble compounds and allow their controlled release in the gastrointestinal tract, which may increase their bioavailability and efficacy. The work of Mehta et al. [67] indicated that microencapsulation allows the concentration of an active ingredient in a product to be increased [68,69]. Thus, microencapsulation can be regarded as a natural extension of the extraction stage—transforming the fragile chemistry of the extract into a stable powder technology ready for application in food, nutraceutical, or pharmaceutical formulations [59].

The most commonly used microencapsulation methods include:

Spray drying—a technique in which a suspension of an extract is sprayed with a carrier (e.g., maltodextrin) in a drying chamber where hot air evaporates water. The resulting powder contains microcapsules with good solubility and stability. It is a fast, cost-effective and easy method to scale up industrially [70,71].

Co-crystallization—a technique in which a bioactive ingredient is deposited together with an excipient (e.g., sugar, polyols) during crystallization. Molecular structures are formed in order to stabilize active compounds, protecting them from oxidation and increasing their shelf-life under storage conditions [68,72].

Ion gelation—a technique that uses a reaction between a polymer (e.g., sodium alginate) and divalent ions (usually calcium). After mixing the extract with the polymer solution, an ion solution is added to the mixture, which leads to the formation of gel microspheres. This technique is particularly useful for encapsulation of aqueous extracts and ensures their high chemical stability [73,74].

The extraction and stabilization techniques described above represent the main methods applicable to various plant materials. Their adaptation to black chokeberry (*Aronia melanocarpa*) and optimization for specific functional food applications are discussed in the following section.

## 6. Aronia Extracts—Methods of Obtaining

Black chokeberry fruits are an excellent raw material for the production of extracts with high biological activity. The choice of the optimal technology should depend on the type of raw material, the chemical properties of the target compounds, and the intended application of the extract. The choice of extraction method depends on the intended form of the final extract, which directly affects its chemical composition and profile of bioactive compounds. The modern extraction techniques discussed enable the recovery of bioactive compounds from various plant tissues naturally rich in these substances, forming the basis for the development of functional foods. According to the literature, not only fruits but also the leaves of black chokeberry bushes, as well as their processed by-products [75,76,77], can be considered valuable sources of polyphenolic components [78,79,80,81]. The future of this field innovation lies in the integration of technology, green chemistry and the personalization of functional ingredients according to consumer needs [82,83,84,85,86].

**Figure 1:**
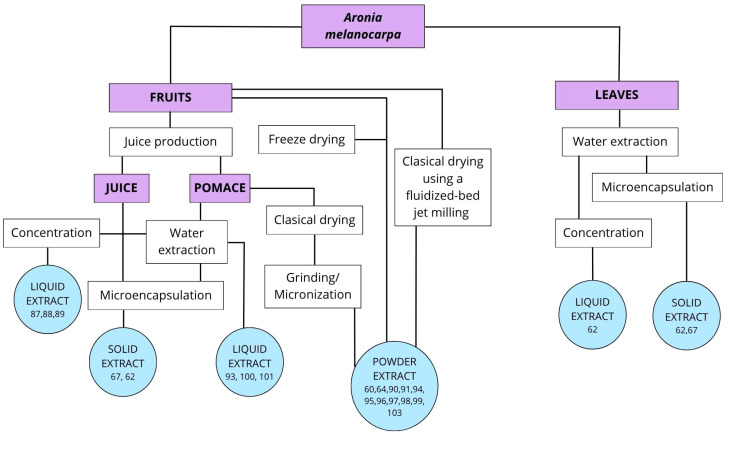
Methods of obtaining different types of black chokeberry extract.

Figure 1 illustrates the main technological schemes for producing aronia-based extracts (from fruits, pomace and leaves) used as components of functional food.

It should be noted that aronia extracts are used not only in the food industry, but also in cosmetics and dietary supplements. By reviewing the literature on this topic, articles were selected that address the topic of using black chokeberry (*Aronia melanocarpa*) fruit extracts in food products.

Table 2 presents a summary of extract types, extraction techniques and their added value in the context of enriching food with bioactive ingredients.

Table 1 summarizes the different extract types described in the literature, their production techniques, and the reported technological and functional effects observed in case studies. The data were compiled based on an analysis of scientific publications from 2016 to 2025. The wide range of extraction and formulation methods applied to *Aronia melanocarpa* demonstrates its versatility as a raw material and its promising potential in the development of modern functional food.

## 7. Application and Stability of Extracts in Processing

Addition of black chokeberry extracts, whether in the form of juice, powder or microcapsules, increases the content of phenolic compounds and the antioxidant potential of food products. The effectiveness of the enrichment operation depends on the type of matrix, the processing technique and the presence of stability aids (e.g., maltodextrin, vegetable proteins, osmotic carriers) [105].

### 7.1. Functional Enrichment and Polyphenol Stability in Aronia-Enriched Foods

The research by Zlabur et al. [64] investigated the effect of adding powdered chokeberry pomace to apple juice, followed by treatment with conventional and high-intensity ultrasound to enhance the release of bioactive compounds from the suspended matrix. The addition of chokeberry pomace powder significantly increased the total polyphenol content from approximately 513 mg GAE/L in the control sample to 955 mg GAE/L in the ultrasound-treated juice. Furthermore, the antioxidant capacity, measured by the ABTS assay, increased from 2165 µmol TE/L to 2250 µmol TE/L after enrichment with chokeberry powder, regardless of the applied ultrasound power.

**Figure 2:**
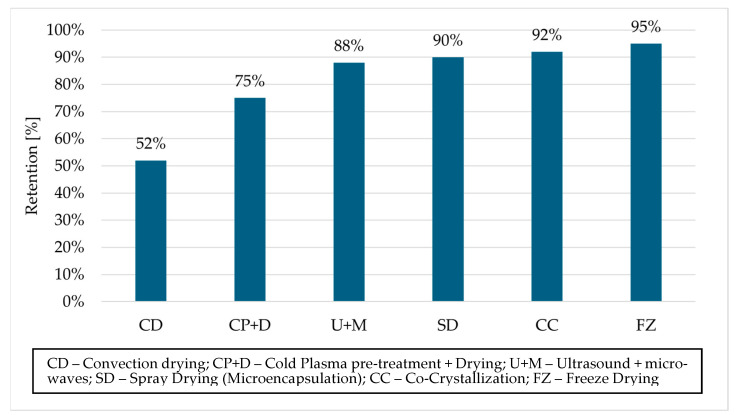
Retention of phenolic compounds (%) in aronia extracts depending on the processing technique used. In-house data based on a literature review [26,63,67].

The stability of phenolic compounds is highly dependent on both the extraction technology used and the method of their preservation. In the study by Do Thi and Hwang [26], the total polyphenol content after drying was reported as 919.7 mg GAE/g in freeze-dried samples, 876.4 mg GAE/g in sun-dried samples, and 792.3 mg GAE/g in oven-dried samples. Although the initial polyphenol content in fresh (undried) samples was not specified, the results clearly indicate that freeze-drying is one of the most effective methods for preserving polyphenols during the drying process. As summarized in Figure 2, literature data confirm a strong dependence of polyphenol retention on drying technique. The results clearly indicate that obtaining an extract rich in polyphenols does not necessarily guarantee the preservation of its quality in the final product.

In the study by Cichowska et al. [106], it was observed that during 12 months of storage at temperatures between 25 and 45 °C, dried apples pre-treated with chokeberry (*Aronia melanocarpa*) concentrate experienced a polyphenol degradation ranging from approximately 40% in freeze-dried samples up to 75% in air-dried samples compared to initial levels before storage. The degradation was more pronounced at higher storage temperatures. Despite these losses, the use of chokeberry concentrate as a pre-treatment effectively enriched the apples with bioactive polyphenols, demonstrating its potential as a natural fortifying agent to enhance the polyphenol content in dried fruit products. However, maintaining optimal storage conditions remains crucial to preserve these health-promoting compounds over time.

### 7.2. Antioxidant and Antimicrobial Roles of Aronia Extracts in Food Preservation

Black chokeberry (*Aronia melanocarpa*) extracts, an exceptionally rich source of polyphenolic compounds, including anthocyanins, procyanidins, and phenolic acids, exhibit strong antioxidant and antimicrobial properties [26]. Their incorporation into food systems not only enhances the functional and nutritional value of the final product, but also contributes to the protection of the food matrix during storage [8].

Numerous studies have shown that chokeberry extracts effectively inhibit oxidative degradation, particularly lipid peroxidation and protein oxidation, processes responsible for quality deterioration in stored food products. For instance, in a study involving pork meat stored under modified atmosphere (MAP) at 4 °C for 29 days, the addition of chokeberry leaf extract resulted in a reduced increase in malondialdehyde (MDA) content—a key marker of lipid oxidation—from ~0.98 mg/kg in the control sample to ~0.85 mg/kg in the treated sample, indicating enhanced oxidative stability [107]. Other research also confirmed the ability of chokeberry polyphenols to inhibit cholesterol oxidation and reduce protein carbonylation in oxidative stress models [108].

Babaoglu et al. [97], on the other hand, conducted trials to use the antioxidant activity of extract from aronia pomace to improve beef oxidative stability, while testing its antimicrobial activity. These studies showed that the presence of aronia extract in beef reduced the level of substances reacting with thiobarbituric acid (TBARS) by 50–60% during cold storage, which proves the strong antioxidant effect of the extract. In addition, these studies demonstrated the effect of aronia pomace extract on the inhibition of the growth of mesophilic aerobic bacteria, the total number of psychrotrophic aerobic bacteria and *Escherichia coli* bacteria. A positive effect on inhibiting the growth of *E. coli* and *Staphylococcus aureus* bacteria was observed in study by Li et al. [109].

Kowalczyk et al. [110] also investigated the beneficial effect of *Aronia* leaf extract on reducing lipid oxidation, showing increased α-tocopherol retention in beef under refrigerated storage conditions. The extract was prepared from dried *Aronia melanocarpa* leaves using hot water extraction (90 °C, 1:10 *m*/*v*) followed by 10 min of ultrasonic treatment. After 30 min of extraction, the infusion was filtered, cooled, frozen, and subsequently lyophilized (−80 °C, 0.04 mbar, 72 h). The lyophilized extract was then used for further analyses and application in the study, where the protective effect on lipids was observed.

In addition to their antioxidant activity, chokeberry extracts have been found to possess antimicrobial properties, which can suppress the growth of spoilage and pathogenic microorganisms. In the same study on pork meat, the application of chokeberry extract led to a notable reduction in microbial load, including *Enterobacteriaceae* and lactic acid bacteria (LAB), particularly during the first 15 days of storage at 4 °C [107]. The antimicrobial effect is attributed to the high content of bioactive phenolics, such as chlorogenic acid, quercetin, and cyanidin-3-O-galactoside, which can disrupt microbial membranes or inhibit key metabolic pathways.

Furthermore, chokeberry extracts have been shown to extend the shelf-life and improve the physical stability of other perishable food products. For example, an edible coating formulated with aronia extract applied to fresh-cut fruits resulted in significantly lower mass loss—by approximately 35.9% at 4 °C and 40.96% at 25 °C—compared to untreated controls during storage [111]. Based on the conducted studies, it can be stated that black chokeberry extract exhibits bacteriostatic activity and effectively extends the shelf-life of fresh apples.

Taken together, these findings clearly demonstrate that the inclusion of chokeberry extracts in food formulations can support long-term preservation by simultaneously limiting oxidative spoilage and microbial growth. This makes them a promising natural alternative to synthetic preservatives in clean-label food production.

### 7.3. Encapsulation and Functional Stabilization Strategies for Aronia Bioactives

The use of aronia extracts in functional foods requires maintaining the stability of bioactive compounds under processing conditions. Among the available strategies, encapsulating bioactive molecules within protective shells appears to be the most effective approach for preserving their structural and functional integrity. Microencapsulation (e.g., by spray drying or ion gelation) allows phenolic compounds to be protected against environmental factors such as oxygen, light, temperature, which significantly affect the degree of preservation of these compounds. Tzatsi and Goula [67] observed that powders obtained by co-crystallization and ion gelation retained approximately 90% of their antioxidant activity after six weeks of storage, indicating a mere ~10% decrease in radical-scavenging capacity under the tested conditions. In their study of encapsulated chokeberry extracts, they also reported that the bulk density of co-crystallized powders ranged between ~0.70 and 0.73 g/cm^3^, which is favourable for reduced oxygen diffusion and improved stability. The authors argue that the crystalline sucrose matrix in co-crystallization reduces the vulnerability of phenolic compounds to degradation (e.g., via oxidation or moisture uptake), thereby contributing to prolonged retention of bioactivity.

Promising results were obtained in the study by Gheorghita et al. [65], where the use of macrogel with aronia juice led to the maintenance of intense colour and high antioxidant activity, ORAC (>5000 μmol TE/100 g) for 30 days. In this case, aronia extract played a double role—as a natural dye and as a strong antioxidant agent. The phenolic compounds contained in it effectively neutralized free radicals, limiting the oxidation and degradation processes of bioactive food ingredients.

Research by Ben-Othman et al. [68] confirmed that spray drying with oilseed proteins as a carrier protected the bioactive components of aronia in plant powders, which were characterized by a high FRAP content (>2.5 mmol Fe^2+^/g) and very good storage stability.

Another approach to stabilization was proposed by Aydogdu Emir et al. [112], who developed innovative antioxidant and visual pH-sensing films based on guar gum incorporated with *Aronia melanocarpa* extract. These films demonstrated effective free radical scavenging capacity alongside stability of anthocyanin pigments under varying environmental conditions. Incorporation of the extract into the polymeric matrix provided synergistic functional properties while protecting the bioactive compounds from oxidative degradation and photodegradation. This approach holds significant potential for active food packaging applications where real-time quality monitoring through colorimetric changes can serve as an indicator of product freshness.

Zhang et al. [113] investigated the stabilization of *Aronia melanocarpa* anthocyanins through complexation with polysaccharides such as carboxymethyl cellulose, pectin, and xanthan gum. The formed anthocyanin-polysaccharide complexes exhibited significantly improved resistance to destabilizing factors including light exposure, high temperature, metal ions, and oxidants. Furthermore, these complexes showed enhanced antioxidant activity and retained biological function in vitro. These findings underscore the efficacy of polysaccharides as stabilizing carriers of bioactive compounds, facilitating their potential applications in the food and pharmaceutical industries where prolonged stability and bioactivity are paramount.

### 7.4. Sensory Impact of Chokeberry Extracts in Food Products

Black chokeberry extracts added to functional foods, in addition to health-promoting benefits and improving technological properties, can affect the sensory properties of the product—both positively and negatively. Features such as colour, taste, aroma, texture and general acceptability are extremely important for the consumer, which translates into the market success of a given product. Therefore, sensory analysis is an important part of the evaluation of innovative products with aronia extracts [97].

A visually attractive and vivid colour is the key elements that determines consumer acceptance of a product. The high content of anthocyanins in black chokeberry fruits (mainly cyanidin-3-galactoside) means that products with the addition of these fruits are characterized by an intense purple-violet colour, which is usually positively received and evaluated by consumers [8]. In the study of Samborska et al. [114], apple slices were subjected to the process of osmotic dehydration using a solution consisting of 65 °Brix of sucrose and 15% aronia juice concentrate (S65-Ch15 determination). Apples dehydrated with aronia concentrate scored the highest sensory scores in all aspects assessed—colour, taste, aroma and overall product quality—compared to the control group treated with only a 65 °Brix solution of sucrose without the addition of fruit concentrate.

The usefulness of aronia extracts as food colourants was studied by Ghendov-Mosan et al. [100], where aronia extract concentrate was used as a natural dye in the production of gummies. Its colouring efficiency was analyzed in comparison with synthetic dyes (E131, E162). It was assessed that aronia extract colouring efficiency was similar to that of control dyes, with a higher assessment of colour attractiveness (average 8.2 out of 9 points). In addition, the use of aronia extract made it possible to increase the antioxidant activity of the gummies (ORAC + 65%).

The introduction of chokeberry extract into the matrix of improved food products results in an increase in astringency in the final product, which is due to the characteristics of the fruit of this species. This is primarily attributed to the presence of procyanidins and tannins. Procyanidins, particularly B-type oligomers, constitute the majority of polyphenolic compounds in chokeberries, accounting for approximately 66% to 82% of the total phenolic content [29]. These compounds are predominantly composed of polymeric units of catechin and epicatechin linked through C-C bonds. Tannins, including hydrolyzable tannins like gallic acid derivatives and condensed tannins, also contribute to the astringent taste [115]. The presence of these compounds is less accepted in products of liquid and semi-liquid consistency, such as beverages. This presents a significant technological challenge in the sensory context. In the study by Petković et al. [116], it was found that beverages that contain more than 15% by volume of aronia juice are too tart and bitter, which resulted in a reduced overall taste experience by more than 25% compared to samples with lower concentrations. For comparison, in the study by Habschied et al. [117], a drink based on barley wort with the addition of aronia juice was produced. Beverages were produced containing 10%, 20%, and 30% chokeberry juice. The formulation with the highest juice content (30%) received the most favourable sensory evaluation; however, further improvements were still required. In order to improve the recipe, it was decided to add peppermint oil and saturate CO_2_. Such a drink received a high score of 8.4 on a 10-point hedonic scale. This study proves that the use of synergistic ingredients (e.g., mint) effectively alleviates the negative sensory aspects of aronia.

In the case of aronia extracts dosed into supplemented products in solid form, it significantly affects the texture of the product. Aronia powders, microcapsules and pomace typically increase the density, viscosity and structure of the matrix. The study by Żbikowska et al. [94] showed that muffins in which wheat flour was replaced with chokeberry pomace flour in an amount not exceeding 10% were characterized by an increase in hardness by 12%, but despite this, the overall acceptability of the texture remained at a high level (average 7.3/9 points). Much better results were obtained for gluten-free baking, which suggests the possibility of using powdered chokeberry pomace in the production of gluten-free baked goods. Gluten-free baked goods with the addition of 10% aronia pomace were characterized by more favourable texture properties (m.in. lower hardness and better moisture), larger baking volume and higher sensory ratings compared to their gluten counterparts with 10% addition of aronia pomace [94]. Also, Szajnar et al. [103] indicated the positive effect of incorporating dried aronia extract as a functional ingredient in the final food product. It was demonstrated that, in fermented dairy products made from sheep’s milk, the addition of aronia fibre increased both the density and viscosity of the product, which was positively evaluated—the average sensory acceptance score reached 8.1 on a 9-point scale.

The addition of black chokeberry in the form of juices or extracts also enriches the aroma profile of food products. Masztalerz et al. [89] conducted an analysis of the profile of volatile aromatic compounds in dried apples subjected to the process of osmotic dehydration in chokeberry-mint solution. An increase in the content of aldehydes and esters responsible for fruity and fresh notes was demonstrated, which correlated with a higher aroma rating in the expert panel’s assessment.

## 8. Future Challenges and Perspectives

The future development of *Aronia melanocarpa* extracts as functional food ingredients faces several technological and formulation challenges. First, sensory barriers such as bitterness, astringency, and, to some extent, intense dark coloration still limit consumer acceptance. Moreover, the variability in raw material composition—resulting from differences in cultivar, ripening stage, and environmental conditions—affects the reproducibility and consistency of product quality [118].

A further concern is that processing operations or formulation strategies designed to mask undesirable sensory characteristics may reduce the bioavailability of polyphenols through matrix interactions with food macromolecules such as proteins, polysaccharides, or lipids. Such effects could ultimately diminish the functional efficacy of the final product and disappoint end users [119].

Collectively, these factors substantially impede the exploitation and commercialization of products and preparations derived from *Aronia* species.

It would be advisable for future research to focus on the development of advanced delivery systems that ensure controlled release and improved bioavailability of health-promoting compounds, as well as stabilization mechanisms that enable the incorporation of aronia extracts into diverse food matrices such as dairy, bakery, and fermented products, while maintaining colour, flavour, and antioxidant activity during processing [120].

Moreover, the valorisation of aronia by-products, such as pomace and leaves, is foreseen to provide a sustainable source of polyphenols consistent with circular economy principles and is expected to play an important role in the future development of the sector. In this context, an integrated approach employing hybrid extraction techniques within processing chains represents a promising direction for future aronia exploitation, enabling maximal recovery of bioactive compounds and the production of tailored, high-value aronia preparations [121].

Increasing attention is also being directed toward personalized functional foods, in which aronia-based ingredients are tailored to specific health benefits and combined with probiotics or dietary fibres to achieve synergistic effects [122].

Another major challenge ahead lies in translating laboratory-scale successes into industrially viable, consumer-accepted, and economically sustainable aronia-based functional foods that retain both their sensory appeal and biological efficacy.

## 9. Conclusions

Black chokeberry extracts are a promising ingredient of functional food, exhibiting a wide spectrum of health-promoting and technological properties. Research conducted in the last decade has shown that their use in food products enables increasing the antioxidant activity, extend shelf-life and improve the visual attractiveness of fortified products. Despite the health benefits of this species, the specific, tart taste of the fruit poses a persistent challenge, limiting its acceptance. Therefore, the key challenge is to optimize the processing technology in terms of sensory composition. A promising approach involves the idea of combining aronia extracts with synergistic plant components.

Since products offered to the market in the functional food category must maintain outstanding bioactive properties with documented health benefits, research is focused on the development of extraction methods that will minimize the degradation of biologically active compounds, while ensuring high process efficiency and thus quality of the final product.

Several advanced extraction techniques, such as ultrasound-assisted extraction (UAE), microwave-assisted extraction (MAE), and supercritical fluid extraction (SFE), have been proven effective in preserving polyphenolic compounds and maintaining the high antioxidant capacity of aronia berries. Moreover, stabilization techniques such as microencapsulation and co-crystallization have been shown not only to enhance the stability of bioactive compounds but also to minimize unfavourable taste characteristics.

The successful implementation of black chokeberry extracts as functional foods ingredients requires further technological, clinical, and sensory research. To fully exploit the potential of chokeberry, it is crucial to identify specific applications in which groups of phytochemicals can remain bioactive without causing adverse sensory effects. Among the most promising categories of functional foods for aronia extracts are beverages (such as antioxidant drinks or herbal infusions), dairy products (e.g., yoghurts, kefirs), and bakery or confectionery products enriched with natural colourants and polyphenols.

Overall, the integration of black chokeberry extracts into well-designed food matrices offers significant potential for developing next-generation functional food that combine health benefits with strong consumer appeal.

## Figures and Tables

**Table 1:** Principal classes of bioactive constituents in black chokeberry (*Aronia melanocarpa* L.) fruits and their relevance to nutritional and physiological functions.

Bioactive Compounds	Approximate Amount in Fruit		Health Benefits and Functions
Anthocyanins [32,33]	600–1000 mg/100 g fresh weight	Cyanidin 3-O-galactoside, Cyanidin 3-O-arabinoside, Cyanidin 3-O-glucoside, Cyanidin 3-O-xyloside	Strong antioxidants; anti-inflammatory; anti-cancer; cardiovascular protection by lowering cholesterol and improving vessel elasticity; improve glucose metabolism; reduce inflammatory markers
Phenolic acids [34]	200–490 mg/100 g fresh weight	Chlorogenic acid (5-O-caffeoylquinic acid), Neochlorogenic acid (3-O-caffeoylquinic acid), dicaffeoylquinic acids	Anti-inflammatory; antioxidant; antibacterial; antidiabetic; DNA protection and chemopreventive effects against cancer; reduce heavy metal toxicity
Flavonols [35]	35–77 mg/100 g fresh weight	Quercetin 3-O-rutinoside, Quercetin 3-O-glucoside, Quercetin 3-O-vicianoside, Isorhamnetin glycosides, Kaempferol derivatives	Potent antioxidants and anti-inflammatory agents; support immune system; promote vascular health
Flavanols [36]	2–3.5 mg/100 g fresh weight	(-)-Epicatechin	Antioxidant; improve glucose metabolism; cardiovascular support
Polyol [32]	~1–2 g/100 g fresh weight	Sorbitol	Acts as a prebiotic; safe for diabetics; supports oral health; mild laxative effect; contributes to sweetness with low glycemic index
Dietary fibre [37,38]	3–8 g/100 g fresh weight	Cellulose (~35%), hemicellulose (~34%), lignins (~24%)	Supports digestive health by promoting beneficial gut bacteria; improves bowel movement; enhances satiety; aids detoxification
Ascorbic acid [36]	8–30 mg/100 g fresh weight	Vitamin C (ascorbic acid)	Strong antioxidant; boosts immune function; supports collagen production; protects cells from oxidative stress

**Table 2:** Characteristics of aronia extracts used for functional food enhancement: product formulation, techniques used, application and technological effects.

Extract Type	Technique	Application	The Added Value of the Enriched Product	References
Aronia juice concentrate/juice	Cold pressing, filtration, pasteurization, concentration (up to 65 Brix)	Osmotic impregnation before drying fruit,	Improved colour (ΔE > 6.0), increased antioxidant capacity (DPPH + 45%); inhibition of anthocyanin degradation by 30% during storage	[87,88,89]
Aronia powder (freeze-dried)	Freeze-drying (−40 °C, 0.1 mbar for 48 h); convection drying (60 °C for 24 h); Pressurized Liquid Extraction, PLE, Microwave-Assisted Extraction, MAE, Supercritical CO_2_ Extraction, scCO_2_.	Sweet confectionery, drinks, dairy desserts, supplements, food additive	Higher polyphenol content (820–900 mg GAE/100 g for freeze-dried vs. 470 mg GAE/100 g for hot air drying); Anthocyanin behaviour above 85%; PLE yielded the highest total phenolics but was less effective for heat-sensitive anthocyanins. MAE achieved a balanced extraction of phenolics and anthocyanins with strong antioxidant activity. scCO_2_ extraction was more selective and eco-friendly but generally yielded lower phenolics than PLE and MAE.	[64,90,91,92]
Aronia pomace	Drying with hot air (50–60 °C); grinding	Bread, snacks, pectin substitutes dairy products	Improvement of fibre content (up to 22% d.m.), reduction in polyphenol losses by up to 10% during baking, increase in moisture retention in baked goods by 15–18%	[93,94,95,96]
Conventional ethanol extract rich in phenolic/polyphenolic compounds	Extraction with 50% ethanol (1:10 *m*/*v*, 60 °C, 30 min); ultrasound-assisted (20 kHz, 30 min)	Oil emulsions, meat, supplements	Total polyphenols (TPC) up to 2400 mg GAE/100 g; reduction in TBARS in meat by 40–60% during storage (14 days, 4 °C)	[60,97,98,99]
Innovative ethanol extract rich in anthocyanin/procyanide	SPE (Solid Phase Extraction) from ethanol and water (50:50), purification on C18 columns. Extraction-adsorption method.	Jelly beans, natural colourants.	Maintaining colour stability (up to 85%) at pH 3–4; inhibition of ascorbic acid oxidation by 52%; colour fastness 28 days at 4 °C. Increased yield and purity of anthocyanin extract produced from chokeberry pomace using a new method compared to the traditional SPE method.	[100,101]
Aronia leaf extract	Hydroalcoholic extraction (60% ethanol, 1:15 *m*/*v*, 40 °C, 2 h), microencapsulation	Meat products	Reduction in lipid oxidation (TBARS) by 42% in beef burgers, increase in sensory acceptability (panel 8/9 pts.)	[62]
Microencapsulated Aronia extracts/Nonencapsulated extract	Spray drying (inlet temperature 170 °C, output temperature 80 °C); co-crystallization with maltodextrin or alginate gelation	Yoghurts, dairy desserts, dietary supplements	Retention of 90–95% of polyphenols after 6 weeks of storage, reduction in Maillard reaction, greater stability at pH 4–5	[67,102]
Aronia powder rich in dietary fibre	Pomace drying (55 °C, 24 h), mechanical separation	Fermented products (e.g., sheep’s milk)	Increase in the number of LAB by 1.5 log CFU/mL; improved texture, increase in overall sensory acceptance	[103]
Macrogels with aronia juice	Gelation of biopolymers (e.g., carboxymethylcellulose, pea protein)	Functional gummies, gelled products	Anthocyanin retention at 80%, improving antioxidant stability, masking astringent taste	[65]
Aronia natura dye extract for replacing synthetic bye E-131, E-162	l Water-ethanol extraction, filtration	Jelly beans, pastries, drinks	Colour fastness for 4 weeks (4 °C, pH 3.0); 65% increase in ORAC of gummies, compliance with “clean label” standards	[93,100]
Fixed oil from berries	Supercritical Fluid Extraction (SFE–CO_2_)	Oil fraction from whole dried *A. melanocarpa* berries	Rich in essential fatty acids (≈70% PUFA: linoleic + α-linolenic), high β-carotene and α-tocopherol—nutritional and cosmetic potential	[79]
Polyphenol-rich extract from pomace	Pressurized Liquid Extraction (PLE) Microwave-Assisted Extraction (MAE) + cryogrinding preprocessing	Recovery of polyphenols and anthocyanins from chokeberry pomace	Higher initial polyphenol concentration; efficient waste recycling; high functional value product Improved behaviour of heat-sensitive compounds; rapid achievement of high concentrations	[92]
Extract from leaves	PLE and MAE	Leaves of *A. melanocarpa* (rather than fruit)	Allows use of leaves as raw material; varies polyphenol profile according to technique—greater flexibility in functional production	[104]

## Data Availability

Not applicable.
